# Supplementary material for: Syndemic violence victimization, alcohol and drug use, and HIV transmission risk behavior among HIV-negative transgender women in India: A cross-sectional, population-based study
Source: PLOS Glob Public Health. 2022 Oct 26;2(10):e0000437. doi: 10.1371/journal.pgph.0000437 (PMC10021466; doi:10.1371/journal.pgph.0000437)
Supplement: S1 Table — All models adjusted for age, education, marital status, sexual identity, forced sex experience during adolescence, HIV risk perception, knowledge of HIV transmission risk, social support, and HIV program exposure. *p < .05, **p < .01, ***p < .001. a Drug use score– 0 to 3. b Alcohol use score– 0 to 7 (consumption in number of days/week). c Violence victimization score– 0 to 5. # Models 1–12: Model 1 –Two-way product term PV x D; Model 2 –Two-way product term PV x A; Model 3 –Two-way product term SV x D; Model 4 –Two-way product term SV x D; Model 5 –Two-way product term PV x SV; Model 6 –Two-way product term A x D; Model 7 –All two-way product terms; Model 8 –All two-way product terms + One three-way product term PV x A x D; Model 9 –All two-way product terms + Two three-way product terms PV x A x D and SV x A x D; Model 10 –All two-way product terms + Three three-way product terms PV x A x D, SV x A x D, and D x PV x SV; Model 11 –All two-way product terms + Three three-way product terms PV x A x D, SV x A x D, and D x PV x SV; Model 12 –All two-way product terms + Three three-way product terms PV x A x D, SV x A x D, D x PV x SV, and A x PV x SV + Four-way product term PV x SV x A x D. $ Models 1–5: Model 1 –Two-way product term VV x D; Model 2 –Two-way product term VV x A; Model 3 –Two-way product term A x D; Model 4 –All two-way product terms; Model 5 –All two-way product terms + Three-way product term VV x A x D. (DOCX) [file pgph.0000437.s001.docx]

**S1 Table. Results of Sensitivity Analyses of the Syndemic Model: Multiplicative Two-/Three-/Four-way Interactions between Physical Violence, Sexual Violence, Drug Use, and Frequent Alcohol Use in Predicting Condomless Anal Sex with Male Partners among HIV-negative Transgender Women (N = 4,607), with Drug Use and Frequent Alcohol Use Specified as Continuous Exposures**

| **Sensitivity analysis 1** | | **Sensitivity analysis 2** | |
| --- | --- | --- | --- |
| **With drug^a^ and alcohol use^b^ as continuous exposures (scores)** | | **With physical and sexual violence as single (violence victimization)^c^ continuous exposure (score)** | |
| **Effects on condomless anal sex** | | | |
| **Significant interactions between adverse psychosocial exposures** | **Significant adjusted OR (95% CI), p value** | **Significant interactions between adverse psychosocial exposures** | **Significant adjusted**  **OR (95% CI), p value** |
| **From total 12 models^#^** | | **From total 5 models^$^** | |
| Model 1: PV x D | 1.88 (1.36, 2.60)*** | Model 1: VV x D | 1.35 (1.16, 1.58)*** |
| Model 7: PV x D | 1.80 (1.22, 2.64)** | Model 4: VV x D | 1.34 (1.11, 1.60)** |
| Model 8: PV x A x D | 1.24 (1.08, 1.43)** | Model 5: VV x A x D | 1.11 (1.03, 1.18)** |
| Model 9: PV x A x D | 1.24 (1.06, 1.44)** | - |  |
| Model 10: PV x A x D | 1.23 (1.05, 1.43)** | - |  |
| Model 11: PV x A x D  A x PV x SV | 1.18 (1.02, 1.37)*  1.39 (1.10, 1.74)** | - |  |
| Model 12: A x PV x SV | 1.31 (1.03, 1.68)* | - |  |

PV, physical violence in the past year; SV, sexual violence in the past year; VV, violence victimization (physical and/or sexual violence); D, drug use in the past year; A, frequent alcohol use in the past week.

All models adjusted for age, education, marital status, sexual identity, forced sex experience during adolescence, HIV risk perception, knowledge of HIV transmission risk, social support, and HIV program exposure.

**p*<.05, ***p*<.01, ****p*<.001.

^a^ Drug use score – 0 to 3.

^b^ Alcohol use score – 0 to 7 (consumption in number of days/week).

^c^ Violence victimization score – 0 to 5.

**^#^** Models 1-12: Model 1 – Two-way product term PV x D; Model 2 – Two-way product term PV x A; Model 3 – Two-way product term SV x D; Model 4 – Two-way product term SV x D; Model 5 – Two-way product term PV x SV; Model 6 – Two-way product term A x D; Model 7 – All two-way product terms; Model 8 – All two-way product terms + One three-way product term PV x A x D; Model 9 – All two-way product terms + Two three-way product terms PV x A x D and SV x A x D; Model 10 – All two-way product terms + Three three-way product terms PV x A x D, SV x A x D, and D x PV x SV; Model 11 – All two-way product terms + Three three-way product terms PV x A x D, SV x A x D, and D x PV x SV; Model 12 – All two-way product terms + Three three-way product terms PV x A x D, SV x A x D, D x PV x SV, and A x PV x SV + Four-way product term PV x SV x A x D.

**^$^** Models 1-5: Model 1 – Two-way product term VV x D; Model 2 – Two-way product term VV x A; Model 3 – Two-way product term A x D; Model 4 – All two-way product terms; Model 5 – All two-way product terms + Three-way product term VV x A x D.
