# Supplementary material for: Syndemic violence victimization, alcohol and drug use, and HIV transmission risk behavior among HIV-negative transgender women in India: A cross-sectional, population-based study
Source: PLOS Glob Public Health. 2022 Oct 26;2(10):e0000437. doi: 10.1371/journal.pgph.0000437 (PMC10021466; doi:10.1371/journal.pgph.0000437)
Supplement: S2 Table — CI: Confidence interval. Adjusted for control variables: Age, education, marital status, sexual identity, forced sex experience during adolescence, HIV risk perception, knowledge of HIV transmission risk, social support, and HIV program exposure. *p < .05, **p < .01, ***p < .001. (DOCX) [file pgph.0000437.s002.docx]

**S2 Table. Results from Testing Rival Theoretical Models of Serially Causal Epidemics and Mutually Causal Epidemics among HIV-negative Transgender Women (N = 4,607)**

| **Hypothesized pathways** | **Direct effect** | **Indirect effect** | **Total effect** |
| --- | --- | --- | --- |
|  | **Estimate (95% CI), p-value** | | |
| **Model of serially causal epidemics:** Drug use and alcohol use as continuous mediators | | | |
| Physical violence on condomless anal sex through: | .40 (.27, .52)*** | .02 (-.01, .04) | .41 (.29, .54)*** |
| Drug use |  | .01 (-.004, .02) |  |
| Frequent alcohol use |  | .01 (-.01, .03) |  |
| Sexual violence on condomless anal sex through: | .08 (-.04, .21) | .04 (.003, .07)* | .12 (-.01, .25) |
| Drug use |  | .03 (-.003, .06) |  |
| Frequent alcohol use |  | .01 (-.01, .03) |  |
| **Model of mutually causal epidemics:** Violence victimization, drug use, alcohol use as continuous exposures | | | |
| Violence victimization on drug use | .18 (.15, .22)*** | **-** | - |
| Drug use on violence victimization | 1.97 (1.38, 2.56)*** | - | - |
| Violence victimization on alcohol use | .44 (.29, .58)*** | **-** | - |
| Drug use on condomless anal sex | .09 (-.01, .20) | **-** | - |
| Alcohol use on condomless anal sex | .01 (-.01, .04) | - | - |
| Violence victimization on condomless anal sex | .22 (.15, .29)*** | **-** | - |

Unstandardized estimates are presented. CI: Confidence interval. Adjusted for control variables: age, education, marital status, sexual identity, forced sex experience during adolescence, HIV risk perception, knowledge HIV transmission risk, social support, and HIV program exposure.

**p*<.05, ***p*<.01, ****p*<.001.
